# Supplementary material for: Multi‐dataset identification of innovative feature genes and molecular mechanisms in keratoconus
Source: J Cell Mol Med. 2024 Sep 19;28(18):e70079. doi: 10.1111/jcmm.70079 (PMC11412914; doi:10.1111/jcmm.70079)
Supplement: Supplementary file 2 — Material S2. [file JCMM-28-e70079-s002.zip › 7.GSEA analysis/1.ARL11/KEGG_NEUROACTIVE_LIGAND_RECEPTOR_INTERACTION.html]

Details for gene set KEGG\_NEUROACTIVE\_LIGAND\_RECEPTOR\_INTERACTION[GSEA]

|  || Dataset | ARL11.group.cls#h\_versus\_l |
| Phenotype | group.cls#h\_versus\_l |
| Upregulated in class | h |
| GeneSet | KEGG\_NEUROACTIVE\_LIGAND\_RECEPTOR\_INTERACTION |
| Enrichment Score (ES) | 0.2648886 |
| Normalized Enrichment Score (NES) | 0.9604382 |
| Nominal p-value | 0.58677685 |
| FDR q-value | 1.0 |
| FWER p-Value | 1.0 |
Table: GSEA Results Summary

  

Fig 1: Enrichment plot: KEGG\_NEUROACTIVE\_LIGAND\_RECEPTOR\_INTERACTION      
 Profile of the Running ES Score & Positions of GeneSet Members on the Rank Ordered List

  

| SYMBOL | TITLE | RANK IN GENE LIST | RANK METRIC SCORE | RUNNING ES | CORE ENRICHMENT || 1 | CHRNB4 | na | 20 | 0.878 | 0.0191 | Yes |
| 2 | GNRHR | na | 24 | 0.851 | 0.0380 | Yes |
| 3 | GZMA | na | 71 | 0.703 | 0.0525 | Yes |
| 4 | GRID1 | na | 138 | 0.617 | 0.0644 | Yes |
| 5 | TRPV1 | na | 190 | 0.586 | 0.0760 | Yes |
| 6 | GABBR2 | na | 193 | 0.583 | 0.0890 | Yes |
| 7 | HTR5A | na | 252 | 0.557 | 0.0998 | Yes |
| 8 | DRD5 | na | 395 | 0.503 | 0.1070 | Yes |
| 9 | CNR2 | na | 574 | 0.461 | 0.1121 | Yes |
| 10 | NPY2R | na | 686 | 0.437 | 0.1187 | Yes |
| 11 | LEP | na | 970 | 0.392 | 0.1193 | Yes |
| 12 | CHRM3 | na | 1116 | 0.374 | 0.1234 | Yes |
| 13 | OPRM1 | na | 1190 | 0.367 | 0.1295 | Yes |
| 14 | C5AR1 | na | 1327 | 0.354 | 0.1335 | Yes |
| 15 | MTNR1A | na | 1331 | 0.354 | 0.1414 | Yes |
| 16 | CHRNA1 | na | 1334 | 0.354 | 0.1492 | Yes |
| 17 | CHRM2 | na | 1385 | 0.349 | 0.1556 | Yes |
| 18 | ADORA1 | na | 1528 | 0.335 | 0.1590 | Yes |
| 19 | GRPR | na | 1540 | 0.334 | 0.1661 | Yes |
| 20 | GRM2 | na | 1611 | 0.328 | 0.1714 | Yes |
| 21 | GRM4 | na | 1776 | 0.313 | 0.1737 | Yes |
| 22 | MC1R | na | 1861 | 0.306 | 0.1781 | Yes |
| 23 | GABRB2 | na | 1882 | 0.305 | 0.1844 | Yes |
| 24 | GABRA2 | na | 2049 | 0.293 | 0.1861 | Yes |
| 25 | HCRTR2 | na | 2128 | 0.287 | 0.1903 | Yes |
| 26 | HTR1A | na | 2225 | 0.280 | 0.1938 | Yes |
| 27 | GRM5 | na | 2391 | 0.271 | 0.1950 | Yes |
| 28 | F2R | na | 2408 | 0.270 | 0.2006 | Yes |
| 29 | CHRNA7 | na | 2437 | 0.269 | 0.2058 | Yes |
| 30 | SSTR4 | na | 2499 | 0.265 | 0.2100 | Yes |
| 31 | CHRNA3 | na | 2684 | 0.256 | 0.2104 | Yes |
| 32 | ADRA2A | na | 2732 | 0.254 | 0.2147 | Yes |
| 33 | GRM6 | na | 2866 | 0.247 | 0.2164 | Yes |
| 34 | P2RX6 | na | 2902 | 0.245 | 0.2208 | Yes |
| 35 | ADORA3 | na | 3008 | 0.240 | 0.2232 | Yes |
| 36 | TRHR | na | 3123 | 0.235 | 0.2251 | Yes |
| 37 | BDKRB2 | na | 3263 | 0.229 | 0.2262 | Yes |
| 38 | CHRNA10 | na | 3330 | 0.225 | 0.2294 | Yes |
| 39 | SCTR | na | 3338 | 0.225 | 0.2342 | Yes |
| 40 | MC4R | na | 3435 | 0.222 | 0.2364 | Yes |
| 41 | GALR2 | na | 3648 | 0.213 | 0.2350 | Yes |
| 42 | CNR1 | na | 3753 | 0.210 | 0.2367 | Yes |
| 43 | GABRB3 | na | 3759 | 0.209 | 0.2412 | Yes |
| 44 | GABRG3 | na | 3851 | 0.206 | 0.2432 | Yes |
| 45 | FPR3 | na | 3862 | 0.205 | 0.2475 | Yes |
| 46 | CHRNA5 | na | 3910 | 0.204 | 0.2507 | Yes |
| 47 | P2RY6 | na | 4090 | 0.198 | 0.2499 | Yes |
| 48 | GABRP | na | 4346 | 0.188 | 0.2467 | Yes |
| 49 | SSTR5 | na | 4365 | 0.187 | 0.2504 | Yes |
| 50 | LTB4R | na | 4405 | 0.186 | 0.2535 | Yes |
| 51 | THRB | na | 4644 | 0.177 | 0.2505 | Yes |
| 52 | GRM7 | na | 4787 | 0.172 | 0.2502 | Yes |
| 53 | HTR6 | na | 4834 | 0.171 | 0.2527 | Yes |
| 54 | S1PR4 | na | 4911 | 0.169 | 0.2543 | Yes |
| 55 | TACR3 | na | 4923 | 0.168 | 0.2578 | Yes |
| 56 | LPAR3 | na | 4925 | 0.168 | 0.2615 | Yes |
| 57 | MC5R | na | 4939 | 0.168 | 0.2649 | Yes |
| 58 | HTR1D | na | 5075 | 0.163 | 0.2646 | No |
| 59 | NPY4R | na | 5257 | 0.158 | 0.2629 | No |
| 60 | S1PR5 | na | 5422 | 0.154 | 0.2616 | No |
| 61 | LPAR1 | na | 5529 | 0.151 | 0.2619 | No |
| 62 | P2RY1 | na | 5613 | 0.148 | 0.2628 | No |
| 63 | NPBWR1 | na | 6324 | 0.128 | 0.2451 | No |
| 64 | GRIK3 | na | 6496 | 0.124 | 0.2429 | No |
| 65 | HCRTR1 | na | 6658 | 0.120 | 0.2409 | No |
| 66 | CCKAR | na | 6738 | 0.118 | 0.2413 | No |
| 67 | HTR2C | na | 6768 | 0.117 | 0.2430 | No |
| 68 | P2RY2 | na | 7054 | 0.111 | 0.2372 | No |
| 69 | GHSR | na | 7176 | 0.108 | 0.2362 | No |
| 70 | P2RY10 | na | 7269 | 0.106 | 0.2359 | No |
| 71 | NTSR1 | na | 7346 | 0.105 | 0.2360 | No |
| 72 | ADCYAP1R1 | na | 7398 | 0.104 | 0.2368 | No |
| 73 | CSH1 | na | 7456 | 0.103 | 0.2375 | No |
| 74 | GLP2R | na | 7518 | 0.101 | 0.2380 | No |
| 75 | NMUR2 | na | 7615 | 0.099 | 0.2374 | No |
| 76 | DRD4 | na | 7813 | 0.096 | 0.2339 | No |
| 77 | GABRA3 | na | 7999 | 0.092 | 0.2306 | No |
| 78 | ADRA1D | na | 8030 | 0.092 | 0.2318 | No |
| 79 | LPAR6 | na | 8151 | 0.089 | 0.2303 | No |
| 80 | CHRNA4 | na | 8485 | 0.083 | 0.2225 | No |
| 81 | GHR | na | 8677 | 0.080 | 0.2187 | No |
| 82 | S1PR3 | na | 8683 | 0.080 | 0.2204 | No |
| 83 | TSHR | na | 8791 | 0.078 | 0.2190 | No |
| 84 | ADRA2C | na | 9072 | 0.074 | 0.2125 | No |
| 85 | F2RL3 | na | 9461 | 0.068 | 0.2028 | No |
| 86 | PLG | na | 9582 | 0.066 | 0.2008 | No |
| 87 | F2RL1 | na | 9658 | 0.065 | 0.2001 | No |
| 88 | RXFP2 | na | 9722 | 0.064 | 0.1997 | No |
| 89 | SSTR3 | na | 9779 | 0.063 | 0.1995 | No |
| 90 | ADRB2 | na | 9848 | 0.062 | 0.1989 | No |
| 91 | NTSR2 | na | 9942 | 0.061 | 0.1975 | No |
| 92 | S1PR1 | na | 10132 | 0.059 | 0.1934 | No |
| 93 | OPRL1 | na | 10158 | 0.058 | 0.1940 | No |
| 94 | GCGR | na | 10282 | 0.057 | 0.1916 | No |
| 95 | FSHR | na | 10393 | 0.055 | 0.1897 | No |
| 96 | GRIN3A | na | 10639 | 0.052 | 0.1837 | No |
| 97 | GABRG2 | na | 10892 | 0.049 | 0.1775 | No |
| 98 | TSPO | na | 11274 | 0.044 | 0.1675 | No |
| 99 | GABRE | na | 11343 | 0.043 | 0.1665 | No |
| 100 | AGTR2 | na | 11576 | 0.041 | 0.1606 | No |
| 101 | CALCRL | na | 11955 | 0.037 | 0.1505 | No |
| 102 | GLRA3 | na | 11984 | 0.036 | 0.1505 | No |
| 103 | PTGDR | na | 12101 | 0.035 | 0.1479 | No |
| 104 | TAAR8 | na | 12651 | 0.030 | 0.1326 | No |
| 105 | GABRB1 | na | 12791 | 0.029 | 0.1293 | No |
| 106 | OPRK1 | na | 12977 | 0.027 | 0.1245 | No |
| 107 | ADORA2B | na | 13467 | 0.023 | 0.1108 | No |
| 108 | GRIK1 | na | 13472 | 0.023 | 0.1112 | No |
| 109 | TAAR9 | na | 13481 | 0.023 | 0.1115 | No |
| 110 | GABRA6 | na | 13615 | 0.022 | 0.1082 | No |
| 111 | GRIN2A | na | 13757 | 0.021 | 0.1045 | No |
| 112 | MLNR | na | 13782 | 0.021 | 0.1043 | No |
| 113 | LTB4R2 | na | 14100 | 0.018 | 0.0955 | No |
| 114 | PTH2R | na | 14300 | 0.017 | 0.0901 | No |
| 115 | RXFP1 | na | 14318 | 0.016 | 0.0900 | No |
| 116 | CYSLTR2 | na | 14931 | 0.012 | 0.0725 | No |
| 117 | HTR7 | na | 15651 | 0.006 | 0.0517 | No |
| 118 | TACR2 | na | 15701 | 0.006 | 0.0504 | No |
| 119 | GPR35 | na | 16391 | 0.001 | 0.0305 | No |
| 120 | GRIK2 | na | 16628 | -0.001 | 0.0236 | No |
| 121 | GRM8 | na | 16641 | -0.001 | 0.0233 | No |
| 122 | C3AR1 | na | 16962 | -0.003 | 0.0141 | No |
| 123 | GH2 | na | 17060 | -0.004 | 0.0114 | No |
| 124 | CYSLTR1 | na | 17094 | -0.004 | 0.0105 | No |
| 125 | GABRR2 | na | 17177 | -0.004 | 0.0082 | No |
| 126 | LHB | na | 17219 | -0.005 | 0.0071 | No |
| 127 | ADORA2A | na | 17446 | -0.006 | 0.0007 | No |
| 128 | CHRNE | na | 17841 | -0.009 | -0.0105 | No |
| 129 | TBXA2R | na | 18286 | -0.012 | -0.0231 | No |
| 130 | GABRG1 | na | 18409 | -0.013 | -0.0264 | No |
| 131 | ADRA1A | na | 18705 | -0.015 | -0.0346 | No |
| 132 | GABRA5 | na | 18742 | -0.015 | -0.0353 | No |
| 133 | TAAR6 | na | 18874 | -0.016 | -0.0387 | No |
| 134 | PRL | na | 18902 | -0.016 | -0.0392 | No |
| 135 | P2RX7 | na | 18916 | -0.016 | -0.0392 | No |
| 136 | GRID2 | na | 19093 | -0.018 | -0.0439 | No |
| 137 | TAAR1 | na | 19377 | -0.020 | -0.0517 | No |
| 138 | PTAFR | na | 19553 | -0.021 | -0.0563 | No |
| 139 | CGA | na | 19555 | -0.021 | -0.0558 | No |
| 140 | DRD3 | na | 19560 | -0.021 | -0.0555 | No |
| 141 | HTR2B | na | 19568 | -0.021 | -0.0552 | No |
| 142 | GRIK4 | na | 19689 | -0.022 | -0.0582 | No |
| 143 | PRLHR | na | 19801 | -0.022 | -0.0609 | No |
| 144 | GALR3 | na | 20344 | -0.026 | -0.0761 | No |
| 145 | NPY1R | na | 20379 | -0.027 | -0.0765 | No |
| 146 | TAAR2 | na | 20388 | -0.027 | -0.0761 | No |
| 147 | HTR1F | na | 20573 | -0.028 | -0.0808 | No |
| 148 | GIPR | na | 20589 | -0.028 | -0.0806 | No |
| 149 | MAS1 | na | 20889 | -0.030 | -0.0886 | No |
| 150 | GRM1 | na | 21083 | -0.032 | -0.0935 | No |
| 151 | BRS3 | na | 21177 | -0.033 | -0.0955 | No |
| 152 | UTS2R | na | 21245 | -0.033 | -0.0967 | No |
| 153 | PARD3 | na | 21300 | -0.034 | -0.0975 | No |
| 154 | PTGER1 | na | 21461 | -0.035 | -0.1014 | No |
| 155 | TAAR5 | na | 21539 | -0.036 | -0.1028 | No |
| 156 | P2RX2 | na | 21624 | -0.036 | -0.1044 | No |
| 157 | ADRB1 | na | 21666 | -0.037 | -0.1048 | No |
| 158 | OPRD1 | na | 21692 | -0.037 | -0.1047 | No |
| 159 | VIPR2 | na | 21712 | -0.037 | -0.1044 | No |
| 160 | TACR1 | na | 21940 | -0.039 | -0.1101 | No |
| 161 | PRSS3 | na | 22001 | -0.040 | -0.1110 | No |
| 162 | NR3C1 | na | 22061 | -0.040 | -0.1118 | No |
| 163 | GABBR1 | na | 22327 | -0.043 | -0.1185 | No |
| 164 | GRIA2 | na | 22338 | -0.043 | -0.1178 | No |
| 165 | MC3R | na | 22349 | -0.044 | -0.1172 | No |
| 166 | THRA | na | 22691 | -0.047 | -0.1260 | No |
| 167 | TSHB | na | 22751 | -0.047 | -0.1266 | No |
| 168 | CTSG | na | 23366 | -0.054 | -0.1432 | No |
| 169 | EDNRB | na | 23504 | -0.056 | -0.1460 | No |
| 170 | CHRNA9 | na | 23612 | -0.057 | -0.1478 | No |
| 171 | CHRNA2 | na | 23787 | -0.060 | -0.1515 | No |
| 172 | HRH1 | na | 23847 | -0.060 | -0.1519 | No |
| 173 | FPR2 | na | 24007 | -0.062 | -0.1551 | No |
| 174 | PTGER4 | na | 24039 | -0.063 | -0.1546 | No |
| 175 | GRIK5 | na | 24220 | -0.065 | -0.1583 | No |
| 176 | CHRNB1 | na | 24257 | -0.066 | -0.1579 | No |
| 177 | GALR1 | na | 24330 | -0.067 | -0.1585 | No |
| 178 | SSTR1 | na | 24502 | -0.069 | -0.1619 | No |
| 179 | LPAR4 | na | 24630 | -0.071 | -0.1640 | No |
| 180 | LPAR2 | na | 24638 | -0.071 | -0.1626 | No |
| 181 | CHRM5 | na | 24993 | -0.076 | -0.1712 | No |
| 182 | VIPR1 | na | 25018 | -0.076 | -0.1702 | No |
| 183 | GRM3 | na | 25083 | -0.077 | -0.1703 | No |
| 184 | GPR156 | na | 25087 | -0.077 | -0.1687 | No |
| 185 | P2RX4 | na | 25171 | -0.078 | -0.1693 | No |
| 186 | P2RX3 | na | 25209 | -0.079 | -0.1686 | No |
| 187 | GABRA4 | na | 25473 | -0.082 | -0.1744 | No |
| 188 | NPBWR2 | na | 25561 | -0.084 | -0.1751 | No |
| 189 | SSTR2 | na | 25727 | -0.086 | -0.1779 | No |
| 190 | BDKRB1 | na | 25851 | -0.088 | -0.1795 | No |
| 191 | GRIA1 | na | 25975 | -0.090 | -0.1811 | No |
| 192 | GLP1R | na | 26008 | -0.090 | -0.1800 | No |
| 193 | LEPR | na | 26569 | -0.100 | -0.1940 | No |
| 194 | GABRQ | na | 26582 | -0.100 | -0.1921 | No |
| 195 | HTR4 | na | 26623 | -0.101 | -0.1910 | No |
| 196 | GLRB | na | 26723 | -0.103 | -0.1916 | No |
| 197 | GRIN2C | na | 26850 | -0.105 | -0.1929 | No |
| 198 | AVPR2 | na | 26924 | -0.107 | -0.1926 | No |
| 199 | CALCR | na | 27001 | -0.108 | -0.1924 | No |
| 200 | CHRM4 | na | 27008 | -0.108 | -0.1901 | No |
| 201 | DRD1 | na | 27274 | -0.114 | -0.1953 | No |
| 202 | CHRNG | na | 27316 | -0.115 | -0.1939 | No |
| 203 | GRIN2D | na | 27406 | -0.117 | -0.1939 | No |
| 204 | HTR1E | na | 27480 | -0.118 | -0.1933 | No |
| 205 | GRIN1 | na | 27606 | -0.121 | -0.1942 | No |
| 206 | HTR1B | na | 27936 | -0.129 | -0.2009 | No |
| 207 | DRD2 | na | 28135 | -0.135 | -0.2036 | No |
| 208 | HRH3 | na | 28253 | -0.138 | -0.2039 | No |
| 209 | P2RY13 | na | 28386 | -0.140 | -0.2046 | No |
| 210 | ADRA2B | na | 28393 | -0.141 | -0.2016 | No |
| 211 | GRIN2B | na | 28410 | -0.141 | -0.1989 | No |
| 212 | S1PR2 | na | 28449 | -0.142 | -0.1969 | No |
| 213 | P2RY14 | na | 28514 | -0.144 | -0.1955 | No |
| 214 | CHRNB2 | na | 28515 | -0.144 | -0.1923 | No |
| 215 | AVPR1B | na | 28803 | -0.152 | -0.1972 | No |
| 216 | PTGER2 | na | 29008 | -0.156 | -0.1996 | No |
| 217 | ADRA1B | na | 29083 | -0.159 | -0.1982 | No |
| 218 | NPFFR1 | na | 29131 | -0.160 | -0.1960 | No |
| 219 | HTR2A | na | 29146 | -0.161 | -0.1928 | No |
| 220 | GLRA2 | na | 29284 | -0.165 | -0.1931 | No |
| 221 | F2 | na | 29479 | -0.172 | -0.1948 | No |
| 222 | NPY5R | na | 29547 | -0.174 | -0.1929 | No |
| 223 | HRH2 | na | 29612 | -0.176 | -0.1908 | No |
| 224 | P2RX5 | na | 29623 | -0.176 | -0.1872 | No |
| 225 | GH1 | na | 29711 | -0.179 | -0.1857 | No |
| 226 | P2RY8 | na | 29798 | -0.181 | -0.1841 | No |
| 227 | APLNR | na | 29934 | -0.186 | -0.1839 | No |
| 228 | FPR1 | na | 30028 | -0.188 | -0.1824 | No |
| 229 | CRHR2 | na | 30151 | -0.192 | -0.1816 | No |
| 230 | GRIA4 | na | 30190 | -0.194 | -0.1784 | No |
| 231 | P2RY4 | na | 30250 | -0.196 | -0.1757 | No |
| 232 | NMBR | na | 30265 | -0.196 | -0.1717 | No |
| 233 | P2RX1 | na | 30312 | -0.198 | -0.1686 | No |
| 234 | CHRNB3 | na | 30692 | -0.211 | -0.1749 | No |
| 235 | AVPR1A | na | 30851 | -0.216 | -0.1746 | No |
| 236 | CHRM1 | na | 31484 | -0.239 | -0.1876 | No |
| 237 | GPR50 | na | 31719 | -0.249 | -0.1888 | No |
| 238 | PTGIR | na | 31874 | -0.255 | -0.1876 | No |
| 239 | AGTR1 | na | 32018 | -0.261 | -0.1859 | No |
| 240 | PTGER3 | na | 32032 | -0.261 | -0.1804 | No |
| 241 | CHRNA6 | na | 32277 | -0.273 | -0.1814 | No |
| 242 | MCHR1 | na | 32352 | -0.278 | -0.1773 | No |
| 243 | CHRND | na | 32474 | -0.285 | -0.1744 | No |
| 244 | GHRHR | na | 32655 | -0.298 | -0.1730 | No |
| 245 | GABRA1 | na | 32830 | -0.310 | -0.1711 | No |
| 246 | CRHR1 | na | 32913 | -0.316 | -0.1664 | No |
| 247 | NPFFR2 | na | 32949 | -0.318 | -0.1603 | No |
| 248 | NMUR1 | na | 33054 | -0.325 | -0.1560 | No |
| 249 | ADRB3 | na | 33080 | -0.328 | -0.1494 | No |
| 250 | EDNRA | na | 33087 | -0.328 | -0.1422 | No |
| 251 | FSHB | na | 33243 | -0.340 | -0.1391 | No |
| 252 | PTGFR | na | 33294 | -0.343 | -0.1328 | No |
| 253 | OXTR | na | 33403 | -0.351 | -0.1281 | No |
| 254 | MCHR2 | na | 33521 | -0.362 | -0.1234 | No |
| 255 | PRLR | na | 33554 | -0.365 | -0.1162 | No |
| 256 | MTNR1B | na | 33644 | -0.374 | -0.1104 | No |
| 257 | KISS1R | na | 33684 | -0.377 | -0.1031 | No |
| 258 | GABRD | na | 33840 | -0.395 | -0.0987 | No |
| 259 | F2RL2 | na | 33846 | -0.396 | -0.0900 | No |
| 260 | GRIN3B | na | 33906 | -0.407 | -0.0826 | No |
| 261 | GPR83 | na | 33938 | -0.410 | -0.0743 | No |
| 262 | MC2R | na | 34007 | -0.420 | -0.0669 | No |
| 263 | GABRR1 | na | 34041 | -0.425 | -0.0583 | No |
| 264 | CCKBR | na | 34232 | -0.460 | -0.0535 | No |
| 265 | PRSS1 | na | 34255 | -0.464 | -0.0438 | No |
| 266 | PTH1R | na | 34273 | -0.468 | -0.0338 | No |
| 267 | HRH4 | na | 34276 | -0.469 | -0.0233 | No |
| 268 | GLRA1 | na | 34356 | -0.487 | -0.0147 | No |
| 269 | GRIA3 | na | 34427 | -0.506 | -0.0054 | No |
| 270 | LHCGR | na | 34633 | -0.630 | 0.0028 | No |
Table: GSEA details [plain text format]

  

Fig 2: KEGG\_NEUROACTIVE\_LIGAND\_RECEPTOR\_INTERACTION      
 Blue-Pink O' Gram in the Space of the Analyzed GeneSet

  

Fig 3: KEGG\_NEUROACTIVE\_LIGAND\_RECEPTOR\_INTERACTION: Random ES distribution      
 Gene set null distribution of ES for **KEGG\_NEUROACTIVE\_LIGAND\_RECEPTOR\_INTERACTION**
